# *In vivo* electrophysiological study of the targeting of 5‐HT_3_ receptor‐expressing cortical interneurons by the multimodal antidepressant, vortioxetine

**DOI:** 10.1111/ejn.15623

**Published:** 2022-03-03

**Authors:** Judith V. Schweimer, Julia T. Brouard, Yan Li, Connie Sánchez, Trevor Sharp

**Affiliations:** ^1^ Department of Pharmacology University of Oxford Oxford UK; ^2^ School of Psychology and Neuroscience University of St Andrews St Andrews UK; ^3^ Lundbeck A/S Valby Denmark; ^4^ Discovery Apellis Pharmaceuticals, Inc Waltham Massachusetts USA; ^5^ Translational Medicine Alkermes, Inc Waltham Massachusetts USA; ^6^ Translational Neuropsychiatry Unit Aarhus University Aarhus Denmark

**Keywords:** dorsal raphe nucleus, electrical stimulation, immunohistochemistry, juxtacellar labelling, serotonin

## Abstract

The antidepressant vortioxetine has high affinity for the ionotropic 5‐HT_3_ receptor (5‐HT_3_R) as well as other targets including the 5‐HT transporter. The procognitive effects of vortioxetine have been linked to altered excitatory:inhibitory balance in cortex. Thus, vortioxetine purportedly inhibits cortical 5‐HT_3_R‐expressing interneurons (5‐HT_3_R‐INs) to disinhibit excitatory pyramidal neurons. The current study determined for the first time the effect of vortioxetine on the *in vivo* firing of putative 5‐HT_3_R‐INs whilst simultaneously recording pyramidal neuron activity using cortical slow‐wave oscillations as a readout. Extracellular single unit and local field potential recordings were made in superficial layers of the prefrontal cortex of urethane‐anaesthetised rats. 5‐HT_3_R‐INs were identified by a short‐latency excitation evoked by electrical stimulation of the dorsal raphe nucleus (DRN). Juxtacellular‐labelling found such neurons had the morphological and immunohistochemical properties of 5‐HT_3_R‐INs: basket cell or bipolar cell morphology, expression of 5‐HT_3_R‐IN markers and parvalbumin‐immunonegative. Vortioxetine inhibited the short‐latency DRN‐evoked excitation of 5‐HT_3_R‐INs and simultaneously decreased cortical slow wave oscillations, indicative of pyramidal neuron activation. Likewise, the 5‐HT_3_R antagonist ondansetron inhibited the short‐latency DRN‐evoked excitation of 5‐HT_3_R‐INs. However unlike vortioxetine, ondansetron did not decrease cortical slow‐wave oscillations, suggesting a dissociation between this effect and inhibition of 5‐HT_3_R‐INs. The 5‐HT reuptake inhibitor escitalopram had no consistent effect on any electrophysiological parameter measured. Overall, the current findings suggest that vortioxetine simultaneously inhibits (DRN‐evoked) 5‐HT_3_R‐INs and excites pyramidal neurons, thereby changing the excitatory:inhibitory balance in cortex. However, under the current experimental conditions, these two effects were dissociable with only the former likely involving a 5‐HT_3_R‐mediated mechanism.

## Graphical abstract

We investigated the effects of the novel antidepressant, vortioxetine, on extracellular single unit recordings from 5‐HT_3_R‐expressing interneurons in the superficial layers of the prefrontal cortex (PFC) of urethane‐anaesthetised rats. 5‐HT_3_R‐interneurons were identified by a short‐latency excitation evoked by electrical stimulation of the dorsal raphe nucleus (DRN). Vortioxetine inhibited the short‐latency DRN‐evoked excitation of 5‐HT_3_R‐interneurons. Juxtacellular labelling found that the putative interneurons had the morphological and immunohistochemical properties of 5‐HT_3_R‐interneurons.

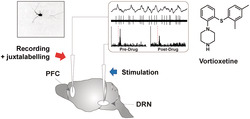

**List of Abbreviations**
5‐HT: 5‐hydroxytryptamine or serotonin
5‐HT_3_R‐ INs: 5‐HT_3_‐receptor expressing interneurons
CB_1_
: Cannabinoid‐1
CCK: Cholecystokinin
COUP TF II: Chicken Ovalbumin Upstream Promoter Transcription Factor II
COV‐IS: coefficient of variance of the interspike interval
ECoG: electrocorticogram
GABA: γ‐aminobutyric acid
LFP: local field potential
PBS: phosphate‐buffered saline
PBS‐X: phosphate‐buffered saline containing Triton‐X
PSTH: peristimulus time histogram
PV: parvalbumin
VGAT: vesicular GABA transporter
VIP: vasointestinal peptide

## 1 INTRODUCTION

Vortioxetine is unusual amongst antidepressant drugs in having high affinity for the excitatory ionotropic 5‐HT_3_ receptor (5‐HT_3_R), as well as the 5‐HT transporter and 5‐HT_1A_, 5‐HT_1B/D_ and 5‐HT_7_ receptors (Sanchez et al., 2015). This makes vortioxetine mechanistically distinct from other antidepressants such as commonly used selective 5‐HT reuptake inhibitors like escitalopram. Moreover, there is evidence from both preclinical studies and studies in patients with major depressive disorder that, in addition to vortioxetine's antidepressant effects, the drug has a procognitive action (McIntyre et al., 2014; Sanchez et al., 2015). This action may be advantageous in depressed patient populations with comorbid cognitive deficits, which are both disabling and often predict poor treatment response (Dunkin et al., 2000; Withall et al., 2011). Although many of vortioxetine's 5‐HT targets likely contribute to its antidepressant effect, the drug's procognitive actions have been linked to the 5‐HT_3_R which has a longstanding association with cognition (Mork et al., 2013; Sanchez et al., 2015; Sharp & Barnes, 2020).

Preclinical evidence suggests that vortioxetine's cognition‐enhancing action involves 5‐HT_3_R blockade (Mork et al., 2013; Pehrson et al., 2016; Sanchez et al., 2015). For example, it is reported that in rodents, procognitive effects of vortioxetine were mimicked by the 5‐HT_3_R antagonist ondansetron and prevented by the 5‐HT_3_R agonist SR57227 (Bétry, Etievant, et al., 2015; du Jardin et al., 2014; Pehrson et al., 2018). Additionally, vortioxetine reversed memory impairment in 5‐HT‐depleted rodents (du Jardin et al., 2014; Jensen et al., 2014), and this effect was recapitulated by ondansetron (Pehrson et al., 2018). A more refined proposal is that the procognitive effects of vortioxetine involve a non‐canonical interaction with the 5‐HT_3_R rather than competitive antagonism *per se* (Riga et al., 2017).

A popular theory is that the cognition‐enhancing properties of vortioxetine are linked to modulation of cortical excitatory: inhibitory balance (Sanchez et al., 2015). Important evidence in favour of this idea is that vortioxetine increased the firing of glutamatergic pyramidal neurons in rat cortex, an effect likely underpinning a simultaneous decrease in cortical slow‐wave oscillations and associated increase in wakefulness (Leiser et al., 2014; Riga et al., 2016, 2017). These responses have been linked to the 5‐HT_3_R blocking action of vortioxetine since ondansetron combined with escitalopram also increased the firing of cortical pyramidal neurons (Riga et al., 2016). In another study of awake rats, ondansetron alone increased cortical oscillation frequency (Leiser et al., 2014). Vortioxetine is hypothesised to activate cortical pyramidal neurons by suppressing inhibitory GABAergic interneurons (INs) (Riga et al., 2016, 2017). In support of this idea, vortioxetine blocked a 5‐HT_3_R‐mediated excitation of putative INs in hippocampus *in vitro* (Dale et al., 2018). Indeed, a major category of cortical (and hippocampal) INs is characterised by expression of the 5‐HT_3_R (Rudy et al., 2011). However, much evidence suggests that different cortical IN categories perform distinct circuit operations. In the case of the 5‐HT_3_R‐INs, it is reported that they preferentially target other cortical IN categories and modulate pyramidal neuron ensembles via disinhibitory mechanisms (Pi et al., 2013). In this scenario, activation and not inhibition of 5‐HT_3_R‐INs would result in pyramidal neuron excitation (Pi et al., 2013). Thus, the precise relationship between the actions of vortioxetine on cortical 5‐HT_3_R‐INs and pyramidal neurons is uncertain.

In the present study we attempted to monitor simultaneously the effects of vortioxetine on cortical 5‐HT_3_R‐INs and pyramidal neurons, and compare actions of vortioxetine with those of ondansetron and escitalopram. A previous *in vivo* electrophysiological study (Puig et al., 2004) reported the detection of presumed 5‐HT_3_R‐INs in superficial layers of the rat prefrontal cortex on the basis their short‐latency excitation by electrical stimulation of the dorsal raphe nucleus (DRN). Here, we adopted this electrophysiological approach and used juxtacellular labelling (Pinault, 1996) to confirm that cortical neurons demonstrating this response to DRN stimulation had chemical and morphological properties in keeping with 5‐HT_3_R‐INs; expressing one or more of a variety of 5‐HT_3_R‐IN markers, including vasoactive intestinal peptide (VIP), cholectocystokinin (CCK) and calretinin, as well as basket or bipolar cell morphology (Kawaguchi & Kubota, 1997; Rudy et al., 2011).

## 2 EXPERIMENTAL PROCEDURES

### 2.1 Animals

Male Sprague–Dawley rats (250–450 g, Harlan) were group‐housed on a 12‐h light/dark cycle with free access to food and water. Experiments were approved by a local ethical review process and the Home Office in accordance with the Animals (Scientific Procedures) Act 1986 (UK) and associated guidelines.

### 2.2 Electrophysiological recordings and juxtacellular labelling

General anaesthesia was induced with isoflurane and then maintained with urethane (1.3–1.5 mg/kg i.p.), plus supplemental doses of ketamine (30 mg/kg, i.m) and xylazine (10 mg/kg, i.m.) as required. Animals were placed in a stereotaxic frame (David Kopf Instruments, Tujunga, CA, USA) and craniotomies were performed prior to implantation of a recording electrode into superficial layers of the prelimbic/infralimbic cortices (coordinates [mm] from bregma: +2.5–3.5 anterioposterior; ±0.5 mediolateral; −2.5 to 4.5 dorsoventral; Paxinos & Watson, 2013) and a stimulating electrode into the DRN (−7.6 to 7.8; 0; 4.5–5.5), respectively. Body temperature was maintained at 37 ± 0.5°C using a homeothermic heating blanket.

Cortical electroencephalogram (ECoG) recordings were made via a steel screw over the left primary motor/somatosensory cortices and referenced against a steel screw positioned over the ipsilateral cerebellum. ECoG data were utilised to ensure that recordings were typically made during slow wave activity which is similar to the predominant brain state during natural sleep. Also, in the presence of urethane cortical slow wave activity can be used as a measure of deep anaesthesia.

Extracellular recordings were made using a glass microelectrode (tip diameter ~1–1.5 μm, 10–30 MΩ) filled with 1.5% Neurobiotin (Vectorlabs, Burlingame, CA, USA) dissolved in 0.5 M NaCl. Electrode signals were direct current‐coupled, amplified (1,000×) and band‐pass filtered (0.3–5 kHz) using a Neurolog system (Digitimer, Welwyn Garden City, UK) and acquired online using a Micro1401 interface and Spike2 software (v8, Cambridge Electronic Design, UK). Mains noise (50 Hz) was eliminated (‘Humbug’ filter). Local field potential (LFP) recordings were acquired via the glass microelectrode and amplified (1,000×) and band‐pass filtered (1–1,000 Hz).

**FIGURE 1:**
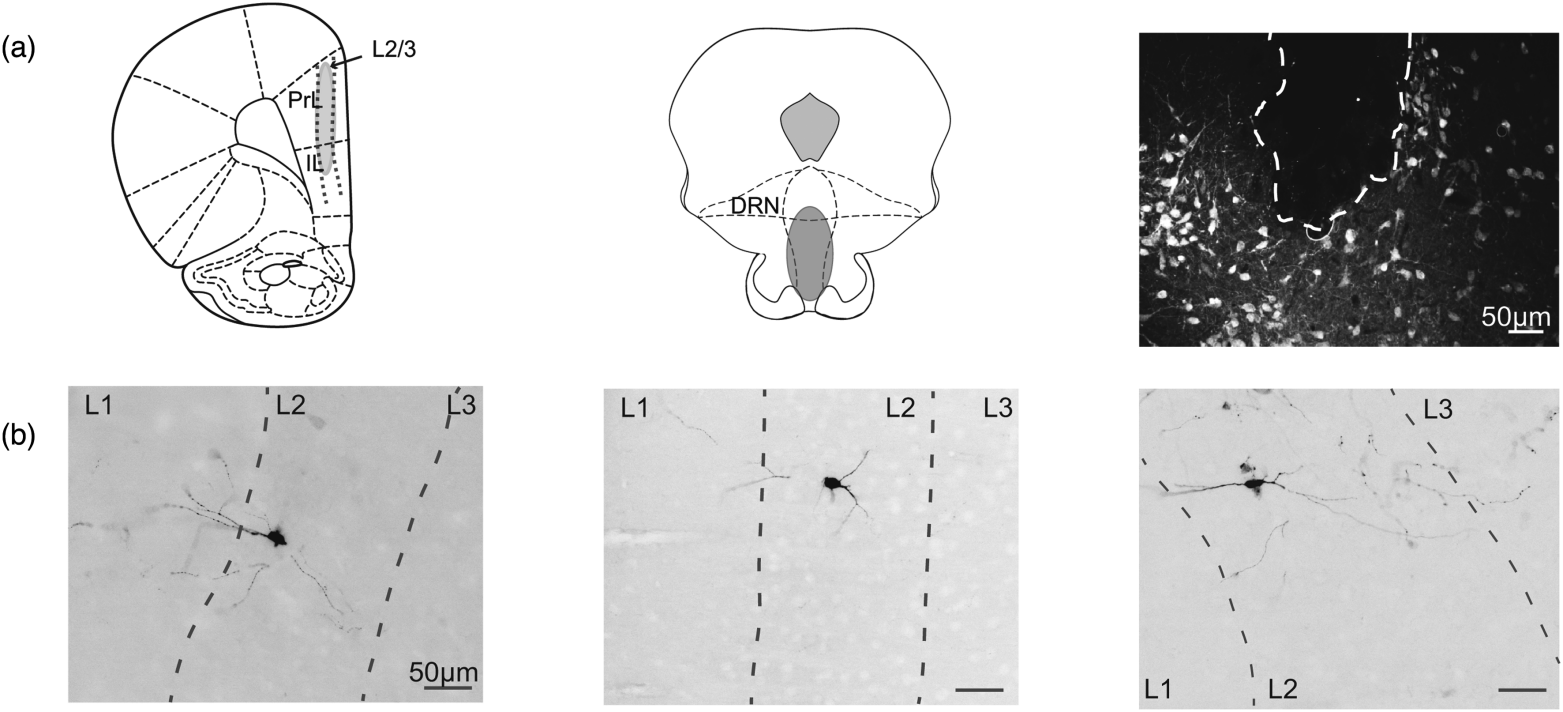
Localisation of recording and stimulation electrodes. (a) Summary of location of labelled neurons and electrode tracts in layer 2/3 of prelimbic/infralimbic cortices (left), stimulation sites within DRN (middle) and example of stimulation electrode in a brain section stained for 5‐HT‐immunoreactivity (right). (b) Examples of juxtacellular‐labelled neurons with non‐pyramidal cell morphology in cortical layers 2/3 (inverted fluorescent images, dotted lines depict cortical layers) that demonstrated a fast excitatory response to DRN stimulation. Plates adapted with permission from Paxinos and Watson (2013)

At the end of recordings in which neurons remained spontaneously active with stable and high amplitude spike waveforms, juxtacellular labelling was attempted as previously described (Allers & Sharp, 2003; Schweimer et al., 2014; Schweimer & Ungless, 2010). Brief positive current pulses (200 ms, 2.5 Hz, 1–10 nA) were applied through the microelectrode and adjusted to obtain modulation of neuronal activity and thereby facilitate uptake of neurobiotin. Examples of successfully juxtacellular‐labelled neurons are shown in Figure 1b.

### 2.3 DRN stimulation

Once single units were encountered and found to be stable the DRN was stimulated (concentric bipolar electrode) over 1–2 min using trains of single, square‐wave current pulses (0.2 ms, 0.5–1.5 mA) applied at 1 Hz. Pulses were delivered by a stimulus generator (Master‐8, AMPI, Jerusalem, Israel) connected to a stimulus isolator (A360, WPI, Sarasota, FL, USA). Putative cortical 5‐HT_3_R‐INs were identified by a fast‐excitatory response (~40 ms latency) to DRN stimulation. In these cases, stimulation was repeated to ensure a stable response before commencing drug administration, and on a further occasion post‐drug. Neurons exhibiting an antidromic response to stimulation were considered as pyramidal neurons projecting to the DRN. At the end of the experiment stimulating electrode placement was verified histologically (Figure 1a).

### 2.4 Drug administration

Vortioxetine lactate, ondansetron hydrochloride dihydrate and escitalopram oxalate were provided by H. Lundbeck A/S (Copenhagen, Valby, Denmark). Vortioxetine was diluted in double‐distilled water, and escitalopram and ondansetron were diluted in isotonic saline.

All drugs were administered via the lateral tail vein in accumulating doses (vortioxetine 0.2, 0.4, 0.8, 1.6 mg/kg; ondansetron 0.32, 0.64 and 1.28 mg/kg; escitalopram 0.1, 0.2, 0.4 and 0.8 mg/kg). Typically one drug was administered per animal with the exception of a small number of animals administered escitalopram followed by ondansetron.

It is previously reported that 0.25 and 1.0 mg/kg i.v. vortioxetine corresponds to 50% and 80% occupancy of the 5‐HT transporter, respectively (Bétry et al., 2013). Accumulated dosing up to 1.6 mg/kg i.v. vortioxetine is likely to give full 5‐HT transporter occupancy. Thus, the vortioxetine dose range in this study covers 5‐HT transporter occupancies achieved with clinically used doses (Sanchez et al., 2015). Unpublished data (Sanchez et al.) show that the escitalopram doses used also cover clinically relevant 5‐HT transporter occupancies.

### 2.5 Immunohistochemistry

Following recordings, animals were transcardially perfused with phosphate‐buffered saline (PBS) followed by 4% paraformaldehyde containing 0.1% glutaraldehyde. Brains were removed and kept in 4% paraformaldehyde/glutaraldehyde overnight before being transferred to a 30% sucrose solution for cryoprotection. Coronal sections (30 μm) were cryostat‐cut and selected sections were then immuno‐stained using a standard protocol for free‐floating sections. Following several rinses in PBS containing 0.2% Triton X‐100 (PBS‐X) sections were incubated in blocking solution (PBS‐X with 10% normal donkey serum) for an hour. Next, sections were washed and incubated in Alexafluor‐488‐conjugated streptavidin (1:1,000, Jackson ImmunoResearch) overnight at 4°C. Sections were then rinsed in PBS and mounted on slides for microscopic examination.

The morphology of juxtacellular‐labelled neurons was initially established prior to imunohistochemical characterisation. Given the current lack of a specific 5‐HT_3_R antibody for immunohistochemical identification of juxtacellular‐labelled 5‐HT_3_R‐INs, sections containing the cell body, dendrites or axons were processed for markers known to be expressed by 5‐HT_3_R‐INs; proCCK, CCK‐8, COUP‐TF II, VIP, calretinin, CB_1_ receptor and VGAT (see Table 1 for details of antibody source, concentrations and labelling outcome). In addition, all labelled neurons were tested for parvalbumin (PV). The choice of markers for a particular neuron was guided by the morphology (e.g., CCK for basket cells). Sections were examined under a epifluorescent microscope and, in many cases, labelling was confirmed using a confocal microscope (LSM 880, Zeiss). Images were cropped to illustrate the region of interest, and brightness and contrast were adjusted using ImageJ64 software (http://imagej.nih.gov/ij/).

**TABLE 1:** Summary of antibodies and immunohistochemical properties of juxtacellular‐labelled cortical neurons demonstrating short‐latency DRN‐evoked excitations

Cell identity	Labelling morphology	PV SySys 195004 (gp) SWANT 235 (m) (1:2000)	CCK‐8 Immunostar 20078 (rb) (1:1,000)	pCCK frontier institute (rb) (1:1000)	CB1 frontier institute (gp) (1:1000)	VIP Immunostar 20077 (rb) (1:1,000)	COUP‐TF II Perseus proteomics (m) (1:500)	Calretinin SWANT 1116 (gt) (1:1000)	VGAT SySys 131004 (gp) (1:1000)
JS250315_1	Basket cell	− (gp)	−	+	+	−	n.t.	n.t.	n.t.
JS010715_1	Double bouquet/horsetail	− (gp)	−	−	−	−	+	+	n.t.
JS080616_1	Double bouquet	− (gp)	−	−	−	−	−	−	+
JS050614_2	Basket cell	− (m)	n.t.	n.t.	n.t.	+	n.t.	n.t.	n.t.
JS050215_1	Soma + proximal dendrites	− (m)	n.t.	n.t.	n.t.	−	n.t.	n.t.	n.t.
JS170615_2	Weakly labelled soma and axon	− (m)	n.t.	n.t.	‐	n.t.	n.t.	n.t.	n.t.
JS120914_1	Soma + proximal dendrites	− (m)	n.t.	n.t.	n.t.	n.t.	n.t.	n.t.	n.t.

Abbreviation: n.t., not tested.

### 2.6 Data analysis

Spike trains were analysed to determine mean firing rate, regularity of firing (coefficient of variation of the inter‐spike‐interval; COV‐IS), and spike waveform duration (time of the onset to first negative trough). To determine the effect of DRN stimulation a peri‐stimulus time histogram (PSTH) was created (0.1 ms time bins) to reveal orthodromic excitation, orthodromic inhibition and antidromic excitation responses. In addition, latency to peak excitation, duration of inhibition and success rate of stimulation (% of stimuli eliciting a spike) were calculated using scripts within Spike2. In the case of fast‐onset orthodromic excitation expected of 5‐HT_3_R‐INs the PSTH typically exhibited a latency to peak of 10–60 ms.

Several neurons had very low baseline firing rate but demonstrated strong responses to DRN stimulation; therefore, drugs were administered during stimulation. Data of these neurons were excluded from drug effect on baseline. The baseline firing rate (for examples of raw traces, see Figures 4a, 5a and 6a) was normalised to pre‐drug values by dividing the values for individuals by the value of the grouped mean and expressed as a %.

To analyse LFP data, power spectra of down‐sampled raw data (1,000 Hz) were created. For this, 60 s bins of raw waveform data were converted using a fast Fourier transform into a power spectrum, and area under the curve was used to quantify the power of the slow wave frequency domain (0.5–1.5 Hz). If a single neuron was lost during drug administration, drug administration was continued to allow for LFP analysis.

Simultaneously collected baseline single unit and LFP data were processed for phase correlation using Spike2 and MATLAB (version 2018B, MathWorks) and the Rayleigh method (Zar, 1999). This analysis was restricted to slow‐wave cortical oscillations (0.5–1.5 Hz). The preferential mean phase of firing during these slow oscillations (circular mean ± s.d.) was determined using custom scripts as described previously (Katona et al., 2017).

### 2.7 Statistical analysis

Baseline firing parameters (firing rate, COV‐IS and action potential width) as well as PSTH readouts (latency of effect and stimulation success rates) were tested for normality (Shapiro–Wilk Test and Q–Q plots) and analysed using ANOVAs (with repeated‐measures as appropriate) and specific post hoc tests as given in the Results section. LFP data were normalised and analysed using repeated measured ANOVAs. Probability values of less than 5% were considered statistically significant. Statistical calculations were carried out using IBM SPSS Statistics for PC Version 25. Mean ± S.E.M values are reported.

## 3 RESULTS

### 3.1 Electrophysiological and morphological properties of cortical neurons

#### 3.1.1 Response to DRN stimulation

**FIGURE 2:**
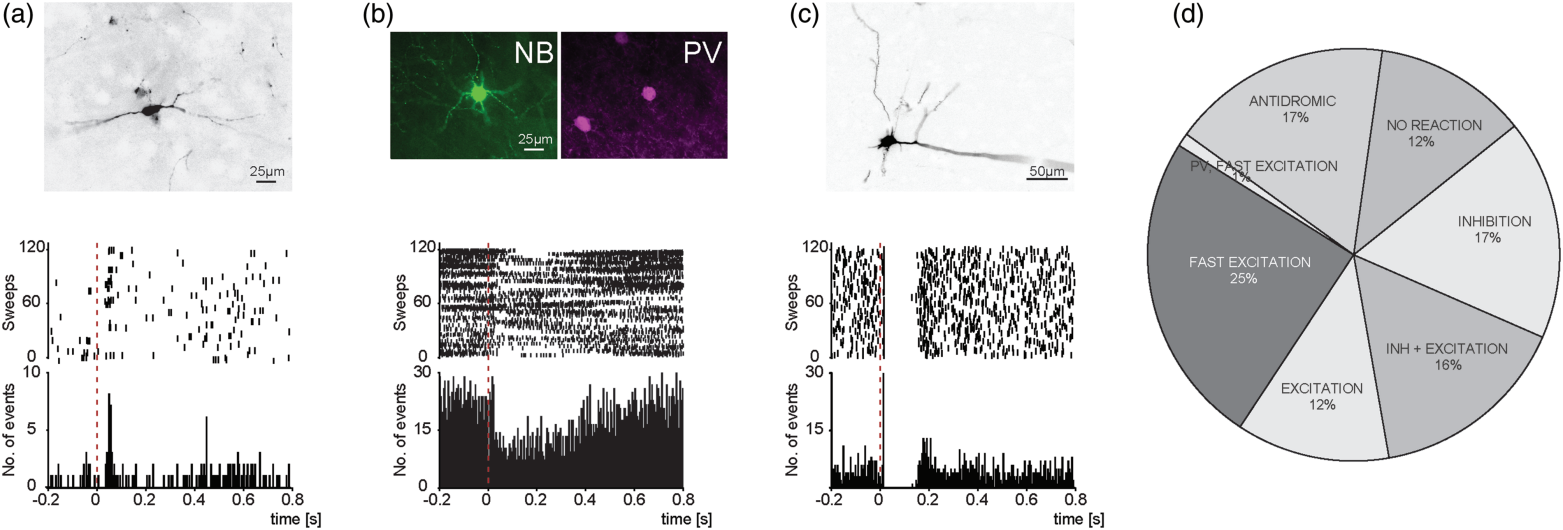
Typical cortical neuron responses to electrical stimulation of the DRN. (a) PSTH (bottom) and raster plot (middle) showing fast excitatory response of a juxtacellular‐labelled putative 5‐HT_3_R‐IN (top). (b) PSTH (bottom) and raster plot (middle) showing slow inhibitory response of a juxtacellular‐labelled, fast‐spiking PV interneuron (top). (c) PSTH (bottom) and raster plot (middle) showing antidromic activation followed by inhibitory response of a juxtacellular‐labelled, pyramidal neuron (top). (d) Summary of responses to DRN stimulation of all neurons tested (*n* = 191) in layers 2/3 of prelimbic/infralimbic cortices

Recordings were obtained from 191 neurons (from 75 rats) located primarily in superficial layers of the prelimbic/infralimbic cortices where 5‐HT_3_R‐INs are most abundant (Lee et al., 2010; Rudy et al., 2011). The majority of these neurons (88.5%) responded to DRN stimulation with responses comprising three main types: (i) short‐latency, antidromic activation (*n* = 34), typically followed by inhibition, (ii) long‐latency orthodromic excitations or inhibitions or both (*n* = 86, and (iii) short‐latency, orthodromic excitation (*n* = 49). Examples of PSTHs demonstrating these responses are shown in Figure 2 together with the proportion neurons showing each type of response (Figure 2d).

Neurons exhibiting a short‐latency, orthodromic excitation were of particular interest as this response was indicative of putative 5‐HT_3_R‐INs in a previous study (Puig et al., 2004). This response had a mean latency of 39.3 ± 1.9 ms (range 9.9–60.8 ms), a mean success rate (ie stimulus: response ratio) of 31.7 ± 3.0%, and the response was stable when the stimulation was repeated. Neurons demonstrating short‐latency, orthodromic excitations had heterogenous baseline firing properties but generally these neurons were slow‐firing (1–2 Hz) and irregular, with biphasic spike waveforms of ~1 ms duration and a firing pattern that was generally in phase with cortical slow wave oscillations (Table 2). These baseline firing properties were distinct from those of juxtacellular‐labelled PV‐positive neurons with a short‐latency excitation (see below) which fired narrow spikes at high frequency. Moreover, none of these neurons demonstrated antidromic spikes that were characteristic of pyramidal neurons. The firing properties of neurons exhibiting these different short‐latency responses are summarised in Table 2.

**TABLE 2:** *In vivo* electrophysiological firing properties of different neuron types in the prefrontal cortex

	Neurons showing short latency activation to DRN stimulation		All neurons					
Neuron type	Latency to respond to DRN stimulation (ms)	Success rate of response to DRN stimulation	Spike waveform width (ms)	Baseline firing rate (Hz)	Regularity (COV)	Angle of phase with slow wave cycles	% of spikes in phase with slow wave cycles	Vector length of firing
5‐HT_3_R‐IN	39.29 ± 1.93 (47)^*^	31.68 ± 2.97 (47)^*^	1.02 ± 0.04 (47)^*^, ^#^	1.79 ± 0.34 (47)	1.25 ± 0.07 (47)	190.43 ± 8.24 (34)^*^	58.11 ± 4.68 (34)	0.49 ± 0.03 (34)
Parvalbumin‐IN	21.38 ± 6.35 (2)	82.51 ± 17.49 (2)	0.64 ± 0.06 (9)^**^	23.40 ± 5.81 (9)^‡^	2.30 ± 0.49 (9)	195.45 ± 14.47 (4)	96.87 ± 1.52 (4)	0.43 ± 0.04 (4)
Pyramidal neuron	16.44 ± 0.99 (34)	91.42 ± 1.60 (34)	1.45 ± 0.07 (34)	1.91 ± 0.33 (34)	1.04 ± 0.03 (34)	223.15 ± 8.91 (25)	67.77 ± 4.68 (25)	0.41 ± 0.03 (25)

*Note*: Properties specific to 5‐HT_3_R‐INs. A short‐latency activation in response to DRN stimulation was common to all 5‐HT_3_R‐INs. On the other hand, parvalbumin‐INs occasionally showed a short‐latency activation but this effect was infrequent and the neurons were easily distinguishable by their fast‐firing of spikes with a narrow waveform. Pyramidal neurons often showed short‐latency excitations but these were antidromic activations. Mean ± SEM (*n*) values are shown.

^*^ *p* < 0.05.

^**^ *p* < 0.01 versus pyramidal cells.

^#^ *p* < 0.05 versus PV‐INs.

^‡^ *p* < 0.05 versus pyramidal cells and 5‐HT_3_R‐INs.

#### 3.1.2 Juxtacellular labelling

Numerous attempts were made to label recorded neurons using the juxtacellular technique (Pinault, 1996). Of the 191 neurons recorded, 28 neurons that responded to DRN stimulation were successfully labelled and all but three neurons were conclusively identified. Fourteen of these labelled neurons were antidromically activated by DRN stimulation and all had the morphology of pyramidal cells as expected (see example in Figure 2c). Moreover, four of the labelled neurons were fast‐firing with narrow spike waveforms and responded preferentially with slow orthodromic inhibition (Figure 2b) or ocassionally with an orthodromic excitation (Table 2), and all were PV‐positive with basket cell or axo‐axonic morphology (see example in Figure 2b; Somogyi et al., 1982).

**FIGURE 3:**
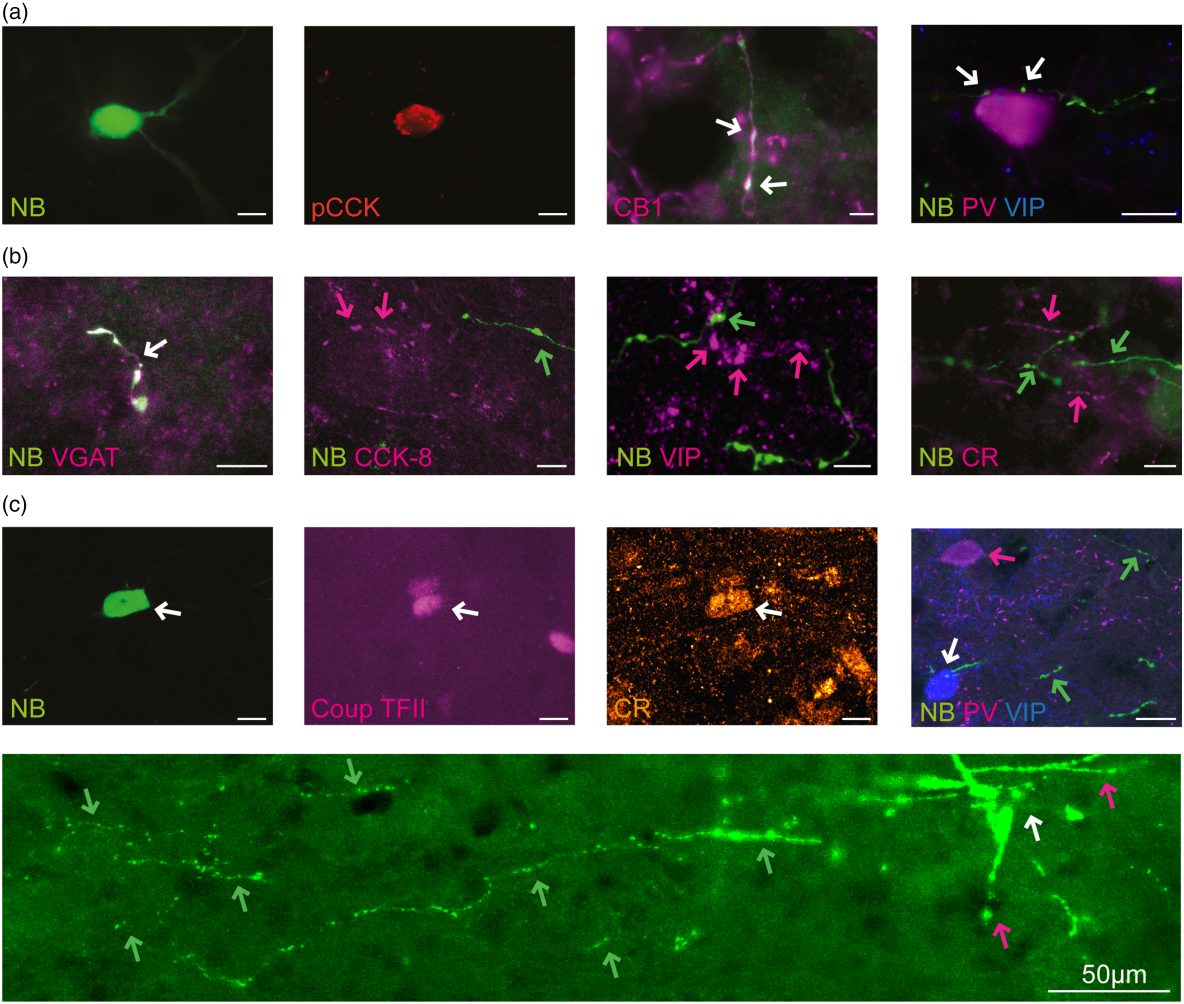
Detailed immunohistochemical analysis of three juxtacellular‐labelled neurons demonstrating fast‐excitatory responses to DRN stimulation. Scale bars are 20 μm. (a) Neurobiotin‐labelled interneuron (green) with basket cell morphology and staining positive for proCCK (red; soma) and CB1 (magenta; axons). This neuron was negative for VIP (blue) and PV (magenta). (b) Neurobiotin‐labelled interneuron (green) with axons (spreading into cortical deeper layers; not shown) staining positive for VGAT and negative for CCK‐8, VIP and calretinin. (c) Neurobiotin‐labelled interneuron (green) staining positive for COUP‐TF II (magenta) and calretinin (orange) but not for VIP (blue) or PV (magenta). This interneuron had a double bouquet neuron morphology and horse‐tail shaped axons spreading into deeper cortical layers. See lower magnification image of one section; axons (green), cell body position (white) and dendrites (magenta). Same interneuron as in Figure 1b on left

Seven neurons that exhibited short‐latency, orthodromic excitation in response to DRN stimulation (Figure 2a) were juxtacellular‐labelled and all had non‐pyramidal morphology including few or no dendritic spines, no apical dendrites and typically with elaborate, local axonal arborisations, indicating that they were interneurons, and all were immunonegative for PV. Moreover, all of these neurons were located in the superficial layers of the prelimbic/infralimbic cortices. Four of these neurons had good somatic and axonal labelling which allowed a detailed immunohistochemical analysis using one or more of a panel of 5‐HT_3_R‐IN markers. One neuron was weakly immunopositive for VIP, and another was immunopositive for the CB_1_ receptor and CCK (Figure 3a), and both neurons were basket cells. For the other two neurons, one was immunopositive for calretinin and COUP‐TF II (Figure 3c) whereas the other was immunonegative for all tested antibodies except for VGAT, confirming its GABAergic nature (Figure 3b). Both of these neurons had a morphology reminiscent of a bipolar or double bouquet cell with axons reaching into the deeper cortical layers in a laminar fashion. The results of the immunohistochemical analysis are summarised in Table 1.

Collectively, the above data confirm that the electrophysiological, immunohistochemical and morphological properties of neurons responding to DRN stimulation with a short‐latency orthodromic excitation are 5‐HT_3_R‐INs, as previously suggested (Puig et al., 2004). The pharmacological properties of these neurons were also in keeping with a 5‐HT_3_R‐IN identity (see below).

### 3.2 Effect of vortioxetine on the firing of putative 5‐HT_3_R‐INs

**FIGURE 4:**
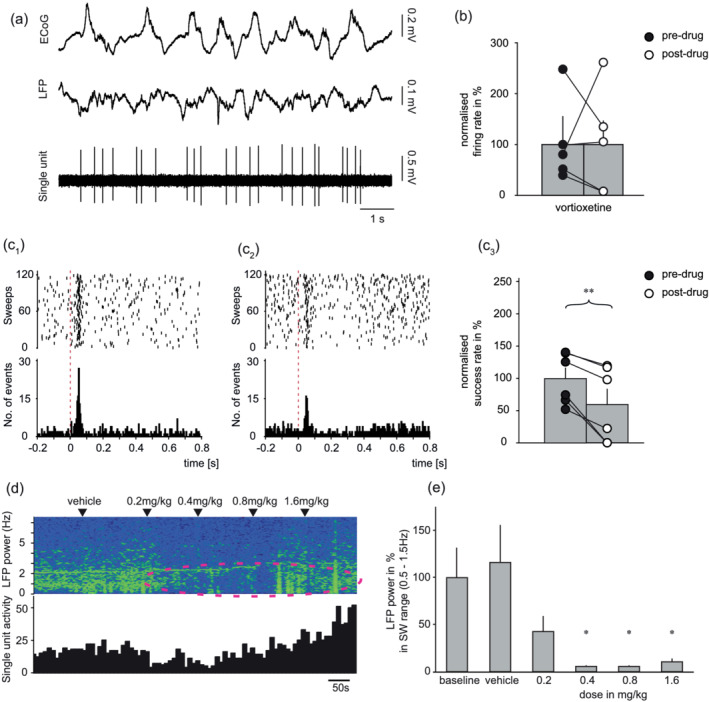
Effect of vortioxetine on neurons exhibiting fast excitatory response to DRN stimulation, and thereby likely 5‐HT_3_R‐INs. (a) Baseline recording of typical neuron during cortical slow oscillations. (b) Group mean ± SEM baseline firing (*n* = 5; not statistically significant, paired *t* test). (c_1_) Example of raster plot and PSTH showing fast‐excitatory response to DRN stimulation before and (c_2_) inhibition after administration of 1.6 mg/kg i.v. vortioxetine). (c_3_) Group mean ± SEM response to DRN stimulation before and after vortioxetine (*n* = 6; *p* < 0.01, paired *t* test). (d) Simultaneous recording of single unit activity (10 s bins) and LFP frequency during cumulative administration of vortioxetine. Note profound shift from slow oscillations to activated brain state without significant changes in baseline firing. (e) Group mean ± SEM change in LFP power (*n* = 7; *p* < 0.05, repeated‐measure ANOVA)

Six neurons (i.e. six rats) demonstrating a short‐latency, orthodromic excitation to DRN stimulation were tested with vortioxetine. Following baseline recording the DRN was initially stimulated and then vortioxetine was administered in accumulating doses (0.2, 0.4, 0.8 and 1.6 mg/kg i.v.), after which the DRN was stimulated a second time. In each case the short‐latency excitatory response to DRN stimulation was reduced by vortioxetine administration (mean effect −40%; paired *t* test, *t* = 4.163, *p* < 0.01; Figure 4c). In these same neurons the effect of vortioxetine on the baseline firing rate was variable with some neurons showing small inhibitions (e.g. see Figure 4b) but overall there was no statistically significant effect.

Simultaneous LFP recordings showed that in all animals tested vortioxetine clearly activated the cortex in a manner consistent with excitation of pyramidal neurons (Riga et al., 2016). Specifically, vortioxetine caused a shift from cortical slow waves to the activated brain state as evident in a reduced power of cortical slow oscillations (0.5–1.5 Hz). This effect of vortioxetine commenced already by the first dose of 0.2 mg/kg and slow wave oscillations were essentially abolished after 0.4 mg/kg (repeated‐measure ANOVA; *F*[1.3, 8.0] = 6.439, *p* < 0.05). The results of an individual animal and the grouped data are shown (Figure 4d,e).

### 3.3 Effect of ondansetron and escitalopram on the firing of putative 5‐HT_3_R‐INs

The selective 5‐HT_3_R antagonist ondansetron and 5‐HT reuptake inhibitor escitalopram were used to explore the involvement of 5‐HT_3_R and 5‐HT reuptake in the effects of vortioxetine.

**FIGURE 5:**
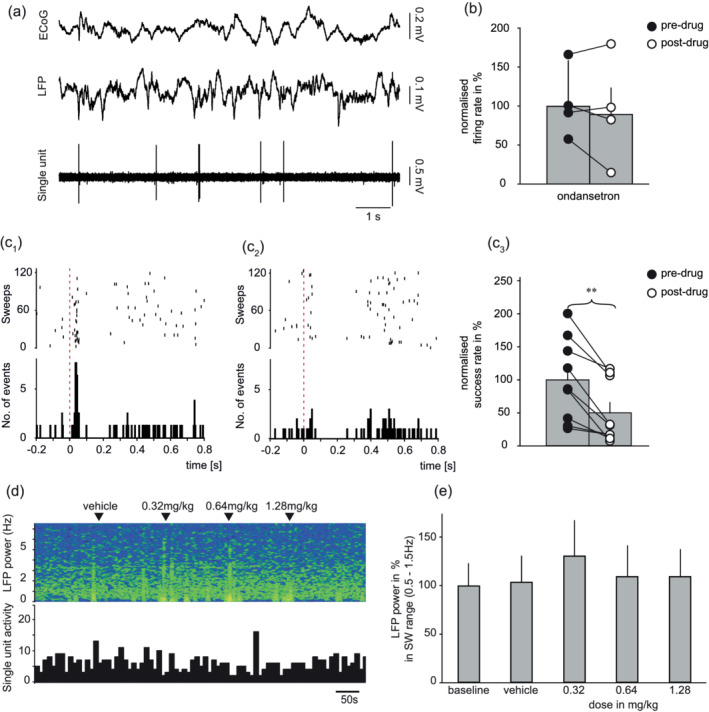
Effect of ondansetron on neurons exhibiting fast excitatory response to DRN stimulation, and thereby likely 5‐HT_3_R‐INs. (a) Baseline recording of typical neuron during cortical slow oscillations. (b) Group mean ± SEM baseline firing (*n* = 5; *p* > 0.05, paired *t* test). (C_1_) Example of raster plot and PSTH showing fast‐excitatory response to DRN stimulation before, and (C_2_) inhibition after administration of 1.28 mg/kg i.v. ondansetron. (C_3_) Group mean ± SEM response to DRN stimulation before and after ondansetron (*n* = 9; *p* < 0.01, paired *t* test). (d) Simultaneous recording of single unit activity (10 s bins) and LFP frequency during cumulative administration of ondansetron. Note lack of change in either LFP or baseline firing. (e) Group mean ± SEM change in LFP power (n = 7; not statistically significant, repeated‐measure ANOVA)

As with vortioxetine, ondansetron in accumulating doses (0.32, 0.64 and 1.28 mg/kg i.v.) inhibited the short‐latency, orthodromic excitation elicited by DRN stimulation (Figure 5c) and this effect was evident in all nine neurons tested (mean effect −49%; paired *t* test, *t* = 4.842, *p* < 0.01). At the same time, ondansetron had no significant effect on the mean baseline firing rate of these neurons (Figure 5b). Additionally, and unlike vortioxetine, in the same animals in which ondansetron inhibited the fast excitatory response to DRN stimulation the drug had no effect on cortical slow wave oscillations (repeated‐measure ANOVA; (*F*[4, 24] = 0.689, not statistically significant; Figure 5d,e).

**FIGURE 6:**
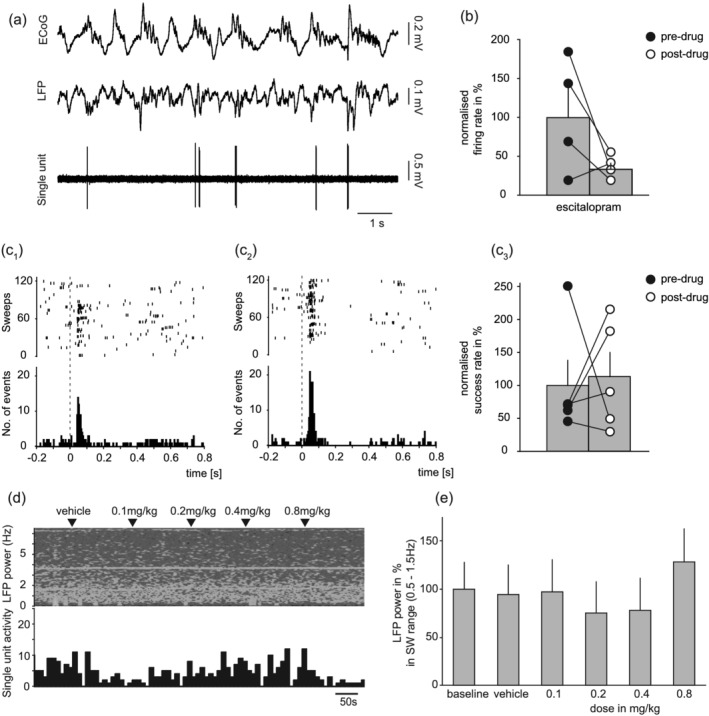
Effect of escitalopram on neurons exhibiting fast excitatory response to DRN stimulation, and thereby likely 5‐HT_3_R‐INs. (a) Baseline recording of typical neuron during cortical slow oscillations. (b) Group mean ± SEM baseline firing (*n* = 4; not statistically significant, paired *t* test). (c_1_) Example of raster plot and PSTH showing fast‐excitatory response to DRN stimulation before and (c_2_) increased response after administration of 0.8 mg/kg i.v. escitalopram. (c_3_) Group mean ± SEM response to DRN stimulation before and after escitalopram (*n* = 5; *p* < 0.01, paired *t* test). (d) Simultaneous recording of single unit activity (10 s bins) and LFP frequency during cumulative administration of ondansetron. Note lack of change in either LFP or baseline firing. (e) Group mean ± SEM change in LFP power (*n* = 6; not statistically significant, repeated‐measure ANOVA)

In contrast to vortioxetine and ondansetron, escitalopram in accumulating doses (0.1, 0.2, 0.4 and 0.8 mg/kg i.v.) had no consistent effect on the short‐latency, orthodromic excitation elicited by DRN stimulation (paired *t* test; *t* = −0.218, not statistically significant, *n* = 5; Figure 6c). Although escitalopram showed a tendency to reduce the baseline firing of these neurons the effect did not reach statistical significance (Figure 6b). Unfortunately, one of these neurons had very low baseline firing activity and could not be included in the final analysis of the baseline data. However, escitalopram had no effect on cortical slow wave oscillations (repeated‐measure ANOVA; (*F*[5, 25] = 1.254, not statistically significant; Figure 6d,e). In three cases escitalopram was co‐administered with ondansetron, and no decrease in the power of cortical slow wave oscillations was observed (data not shown).

## 4 DISCUSSION

A current theory is that a likely mechanism underlying the procognitive effects of the antidepressant vortioxetine is the targeting of cortical interneurons from the 5‐HT_3_R‐IN class, resulting in the disinhibition of excitatory pyramidal neurons (Sanchez et al., 2015). However, to date, the effect of vortioxetine on 5‐HT_3_R‐INs has not been tested *in vivo*. Moreover, the effects of vortioxetine 5‐HT_3_R‐INs and pyramidal neurons have not been monitored side by side. Here, we used *in vivo* electrophysiological approaches to detect 5‐HT_3_R‐INs (DRN‐evoked excitation) whilst simultaneously monitoring cortical oscillations as a surrogate marker of pyramidal neuron activity. The main findings were; (i) a subset of cortical neurons responded to DRN stimulation with a short‐latency, orthodromic excitation as reported previously (Puig et al., 2004), (ii) juxtacellular labelling revealed that neurons exhibiting this response to DRN stimulation had diverse morphological and immunochemical properties albeit typical of 5‐HT_3_R‐INs, (iii) in keeping with the latter result vortioxetine inhibited this short‐latency excitation as did the more specific 5‐HT_3_R antagonist ondansetron, suggesting a 5‐HT_3_R‐mediated mechanism, (iv) neither vortioxetine nor ondansetron had a consistent effect on the baseline firing of 5‐HT_3_R‐INs and, (v) vortioxetine but not ondansetron (or escitalopram) activated the cortex (i.e. decreased slow wave oscillations) reminiscent of pyramidal neuron activation, and suggesting a dissociation between changes in 5‐HT_3_R‐IN and pyramidal neuron function.

### 4.1 Identification of 5‐HT_3_R‐INs

The present study demonstrated that a population of cortical neurons respond to electrical stimulation of the DRN with a short‐latency excitation, thereby confirming previous findings (Puig et al., 2004). Several of our findings strongly support the idea proposed by Puig and colleagues that these neurons are 5‐HT_3_R‐INs. These neurons were predominantly located in superficial layers of the prefrontal cortex where 5‐HT_3_R‐INs are most abundant (Lee et al., 2010; Rudy et al., 2011). Moreover, as predicted from electrophysiological studies of 5‐HT_3_R‐INs *in vitro* (Ferezou et al., 2002; Kubota, 2014), these neurons had slow and irregular baseline firing properties and were easily distinguishable from both PV‐INs (fast‐firing, narrow spikes, mixed DRN‐evoked orthodromic activation and inhibition) and pyramidal neurons (DRN‐evoked antidromically activation). It is reported that DRN stimulation evoked a relatively slow orthodromic excitation of fast‐spiking neurons (Puig et al., 2010) mediated by 5‐HT_2A_ receptors (Puig et al., 2010). Here, in one example, the very short‐latency activation of a PV‐IN was not blocked by vortioxetine (data not shown) and putatively was mediated by co‐released glutamate (see below).

Importantly, all slow‐firing, juxtacellular‐labelled neurons which exhibited a fast‐excitatory response to DRN stimulation had morphological, immunohistochemical and pharmacological properties expected of 5‐HT_3_R‐INs. Thus, these neurons were morphologically classified as interneurons, having basket cell or bipolar/double bouquet cell appearance as reported for 5‐HT_3_R‐INs (Lee et al., 2010, Rudy et al., 2011). There is no good 5‐HT_3_R antibody currently available but all neurons exhibited immunohistochemical labelling consistent with a 5‐HT_3_R‐IN identity, although clear heterogeneity was evident as expected from previous studies (Kubota, 2014; Lee et al., 2010; Morales & Bloom, 1997; Rudy et al., 2011). Thus, individual neurons demonstrated labelling for CCK, CB_1_ receptor, VIP, COUP‐TF II, calretinin or VGAT and, importantly, all were immunonegative for PV. Although three of these neurons were immunonegative for VIP, a subpopulation of 5‐HT_3_R‐INs that lacks VIP expression is reported (Kubota, 2014, Rudy et al., 2011). Furthermore, we cannot exclude that these neurons were false negative for VIP due to low antigen levels.

The excitatory response DRN stimulation studied here was orthodromic, short onset (~39 ms) and short duration (~50 ms), which is consistent with an ionotropic receptor‐mediated response. Indeed, the response was sufficiently robust and reproducible to allow pharmacological testing and shown to be inhibited by the selective 5‐HT_3_R antagonist, ondansetron. This result agrees with a previous study that found the same response to be inhibited by ondansetron as well as the 5‐HT_3_R antagonist tropisetron (Puig et al., 2004). Although earlier *in vitro* studies reported desensitisation of cortical 5‐HT_3_R‐INs to application of exogenous 5‐HT (Zhou & Hablitz, 1999), this was not detected here nor in the earlier study (Puig et al., 2004). It is possible that this desensitisation related to run down of cation gradients that does not occur under the *in vivo* conditions used in the present study. Interestingly, although ondansetron almost completely blocked DRN‐evoked fast excitations in some neurons, in other neurons, the blockade was partial.

This partial blockade might in part be explained by an incomplete occupancy of 5‐HT_3_Rs by ondansetron at the doses used (0.32–1.28 mg/kg i.v.). Thus, ondansetron is a potent P‐glycoprotein substrate, and a previous study reported 40% 5‐HT_3_R occupancy with 1 mg/kg ondansetron and that higher occupancy could not be achieved by increasing the dose further (Bétry, Overstreet, et al., 2015). Alternatively, the involvement of other excitatory 5‐HT receptors in this excitatory response to DRN stimulation cannot be excluded. Some cortical neurons (likely pyramidal neurons) respond to DRN stimulation with an orthodromic excitatory response (Hajós et al., 2003; Puig et al., 2004, 2005); however, this response is of relatively long duration and likely mediated by metabotropic 5‐HT_2A_ receptors. Recently, the co‐release of glutamate from 5‐HT neurons was reported to induce fast excitatory responses in amygdala mediated by ionotropic glutamate receptors (Sengupta et al., 2017). Therefore, it is a possibility that co‐released glutamate contributes to DRN‐evoked excitation and accounts for the incomplete blockade by ondansetron.

### 4.2 Effect of vortioxetine on 5‐HT_3_R‐INs

As with ondansetron, vortioxetine also inhibited the fast‐excitatory effect of DRN stimulation which is consistent with the 5‐HT_3_R antagonist properties of this drug (Sanchez et al., 2015) and also recent studies finding that vortioxetine blocked both 5‐HT‐ and 5‐HT agonist‐evoked excitation of putative 5‐HT_3_R‐INs in hippocampal slices (Dale et al., 2018). The current *in vivo* data are the first evidence that vortioxetine targets 5‐HT_3_R‐INs in the prefrontal cortex when administered systemically, thereby adding plausibility to the view that this effect may contribute to the actions of the drug in behavioural models (Sanchez et al., 2015).

Interestingly, vortioxetine had no clear‐cut inhibitory effect on the baseline firing of neurons in which the fast‐excitatory effect of DRN stimulation was clearly reduced. This said, the baseline firing rate showed a large variability between neurons and the conclusions regarding treatment effects should be treated cautiously. Two neurons showed evidence of decreased firing after vortioxetine which might reflect heterogeneity in the 5‐HT_3_R‐IN population (see above), and it is possible that an increased sample size would reveal a more consistent effect. On the other hand, ondansetron also had no overall inhibitory effect on the baseline firing of 5‐HT_3_R‐INs suggesting that there is a general lack of 5‐HT tone on 5‐HT_3_R‐INs under the present conditions.

In the same experiments in which vortioxetine inhibited the fast excitatory response to DRN stimulation, LFP recordings were obtained in the prefrontal cortex. It was apparent from these recordings that vortioxetine caused a striking switch from slow wave oscillations to the activated brain state as previously observed in awake rats (Leiser et al., 2014), consistent with increased firing of pyramidal neurons. Indeed, it was reported that similar doses of vortioxetine to those used here increased the firing of individual pyramidal neurons in the cortex (Riga et al., 2016). This shift to the activated brain state is similar to shifts from slow oscillations to REM sleep or awakening from natural sleep (see Clement et al., 2008). These results are in line with increases of wakefulness after vortioxetine administration to behaving animals, accompanied with an increase of theta activity indicating an increase in active behaviour (Leiser et al., 2014). Moreover, cortical activation has been linked to the procognitive effects of vortioxetine in rodents and humans (McIntyre et al., 2014; Pehrson et al., 2018; Sanchez et al., 2015).

It is hypothesised that cortical activation by vortioxetine is linked to 5‐HT_3_R blockade, inhibition of 5‐HT_3_R‐INs and thereby disinhibition of cortical pyramidal neurons (Leiser et al., 2014; Riga et al., 2016; Sanchez et al., 2015). The current data suggest that this sequence of events may be an over‐simplification. Thus, despite inhibiting the fast‐excitatory responses of 5‐HT_3_R‐INs to DRN stimulation, neither vortioxetine nor ondansetron consistently reduced the baseline firing of these neurons. On the other hand, vortioxetine but not ondansetron evoked cortical activation. Collectively, these data suggest that an action on 5‐HT_3_R‐INs is not a requirement for cortical activation. Moreover, if vortioxetine causes disinhibition of cortical circuits to bring about cortical activation, it is unlikely to involve an action on 5‐HT_3_R‐INs. The latter idea would be consistent with emerging evidence that the preferential target of 5‐HT_3_R‐INs is other interneuron populations rather than pyramidal neurons (Pi et al., 2013). Thus, the current data support a more nuanced scenario in which vortioxetine targets 5‐HT_3_R‐INs to bring about subtle changes in the timing of events within the cortical microcircuit that alters the processing of incoming information. This then interacts with enhanced pyramidal neuron excitability to bring about improvements in cognition.

The current study did not fully investigate the pharmacological mechanism underlying the increase in pyramidal neuron excitability induced by vortioxetine which may ultimately link to the procognitive effects of the drug. However, the lack of effect of ondansetron or escitalopram (alone or combined) argues against the involvement of blockade of the 5‐HT_3_R or 5‐HT transporter. Vortioxetine also has affinity for 5‐HT_1A_, 5‐HT_1B/D_ and 5‐HT_7_ receptors, and the drug is likely to have meaningful occupancies at these sites when administered to the rat in the i.v. doses used here (Mork et al., 2013). An action at one or more of these targets may contribute to the increase in firing of cortical pyramidal neurons and thus a procognitive action. Interestingly, 5‐HT_1A_ receptor agonists are reported to activate pyramidal neurons in frontal cortex (e.g., Hajós et al., 1999) and have procognitive effects in animal models (see Mork et al., 2013).

Finally, it should be noted that the current experiments were carried out under anaesthetised conditions and that under awake conditions there may be a higher 5‐HT tone on 5‐HT_3_R‐INs. Thus, vortioxetine may indeed decrease the baseline firing of these neurons in such circumstances. Future technically challenging experiments investigating the effect of vortioxetine on the baseline firing of 5‐HT_3_R‐INs in awake animals would help resolve these issues.

## 5 CONCLUSIONS

The current study provides the first evidence that vortioxetine targets 5‐HT_3_R‐INs when administered systemically, and that this effect occurs simultaneous to increased excitability of pyramidal neurons to change excitatory:inhibitory balance in cortex. However, experiments with ondansetron suggest that these two effects are dissociable with only the former likely involving a 5‐HT_3_R‐mediated mechanism.

## CONFLICT OF INTEREST

CS and YL were full‐time employees at H. Lundbeck A/S.

## AUTHOR CONTRIBUTIONS

JVS contributed to the study design, data collection, data analysis, wrote paper; JTB collected and analysed data; YL, CS gave advice on experimental design, revised manuscript; TS contributed to the study design, wrote paper. All authors have read and approved the final manuscript.

### PEER REVIEW

The peer review history for this article is available at https://publons.com/publon/10.1111/ejn.15623.

## Data Availability

The data that support the findings of this study are available from the corresponding authors, JVS and TS, upon request.
